# Reducing Deaths from Severe Pneumonia in Children in Malawi by Improving Delivery of Pneumonia Case Management

**DOI:** 10.1371/journal.pone.0102955

**Published:** 2014-07-22

**Authors:** Penelope M. Enarson, Robert P. Gie, Charles C. Mwansambo, Ellubey R. Maganga, Carl J. Lombard, Donald A. Enarson, Stephen M. Graham

**Affiliations:** 1 Child Lung Health Division, International Union Against Tuberculosis and Lung Disease, Paris, France; 2 Desmond Tutu TB Centre, Department of Paediatrics and Child Health, Faculty of Medicine and Health Sciences, Stellenbosch University, Tygerberg, South Africa; 3 Department of Paediatrics and Child Health, Faculty of Medicine and Health Sciences, University of Stellenbosch, Tygerberg, South Africa; 4 Ministry of Health, Lilongwe, Malawi; 5 UNICEF Malawi, Lilongwe, Malawi; 6 Biostatistics Unit, South Africa Medical Research Council (MRC), Cape Town, South Africa; 7 Centre for International Child Health, University of Melbourne Department of Paediatrics and Murdoch Children's Research Institute, Royal Children's Hospital, Melbourne, Australia; London School of Hygiene and Tropical Medicine, United Kingdom

## Abstract

**Objective:**

To evaluate the pneumonia specific case fatality rate over time following the implementation of a Child Lung Health Programme (CLHP) within the existing government health services in Malawi to improve delivery of pneumonia case management.

**Methods:**

A prospective, nationwide public health intervention was studied to evaluate the impact on pneumonia specific case fatality rate (CFR) in infants and young children (0 to 59 months of age) following the implementation of the CLHP. The implementation was step-wise from October 1^st^ 2000 until 31^st^ December 2005 within paediatric inpatient wards in 24 of 25 district hospitals in Malawi. Data analysis compared recorded outcomes in the first three months of the intervention (the control period) to the period after that, looking at trend over time and variation by calendar month, age group, severity of disease and region of the country. The analysis was repeated standardizing the follow-up period by using only the first 15 months after implementation at each district hospital.

**Findings:**

Following implementation, 47,228 children were admitted to hospital for severe/very severe pneumonia with an overall CFR of 9•8%. In both analyses, the highest CFR was in the children 2 to 11 months, and those with very severe pneumonia. The majority (64%) of cases, 2–59 months, had severe pneumonia. In this group there was a significant effect of the intervention Odds Ratio (OR) 0•70 (95%CI: 0•50–0•98); p = 0•036), while in the same age group children treated for very severe pneumonia there was no interventional benefit (OR 0•97 (95%CI: 0•72–1•30); p = 0•8). No benefit was observed for neonates (OR 0•83 (95%CI: 0•56–1•22); p = 0•335).

**Conclusions:**

The nationwide implementation of the CLHP significantly reduced CFR in Malawian infants and children (2–59 months) treated for severe pneumonia. Reasons for the lack of benefit for neonates, infants and children with very severe pneumonia requires further research.

## Introduction

Pneumonia is the most frequent cause of death in children less than five years of age [1]. In sub-Saharan Africa, child pneumonia deaths account for an estimated 18% of under-five mortality of which three per cent occur in the neonatal period [1]. The incidence of pneumonia and the case-fatality rate are highest among infants and decline with increasing age [2].

In 1984, the World Health Organization (WHO) introduced standardized case-management (SCM) of pneumonia [3] that became an important part of integrated child health programmes and WHO recommended approaches in clinical care [4]. Pneumonia-related deaths declined following the introduction of community-based SCM [5]. Hospitalized cases represent the more severe spectrum of disease and are more likely to die. The effectiveness of hospital-based SCM on pneumonia-related mortality has not been reported and there are no studies of the effectiveness of nation-wide, hospital-based programmes within existing health services.

The International Union Against Tuberculosis and Lung Disease (The Union) developed the Child Lung Health Programme (CLHP) for SCM of pneumonia in hospitalized children based on its model for tuberculosis services. The Union model for tuberculosis was adopted by the WHO in 1993 as the basis of its global strategy for control of tuberculosis (the DOTS strategy) [6] which the World Bank assessed as among the most cost-effective health interventions in low-income countries [7]. The model includes accurate accounting for services, materials, and training and permits management of supplies to ensure no disruption of essential therapies. The Ministry of Health (MoH), Government of Malawi, requested assistance from The Union to improve the management and outcome of pneumonia in Malawian children. The Union assisted the Ministry of Health to adapt and implement the CLHP within the country's existing health services to ensure sustainability. The model and its implementation have been previously described [8].

The aim of this study was to assess the benefits of this strategy by measuring the trend over time in the case fatality rate (CFR) in neonates, infants and young children (one week to 59 months of age) hospitalized with severe or very severe pneumonia.

## Methods

### Ethics Statement

The CLHP was routine patient management and data from it was routinely collected. The data used for the study was aggregated data from Monthly Recording Forms - no individual identification appeared on these reports. Permission to use the data was received from the Ministry of Health, Republic of Malawi. Approval to analyze the data was obtained from the Union's Ethical Advisory Group (EAG: 05/10) and the Human Research Ethics Committee (N10/09/285), Faculty of Medicine and Health Sciences, Stellenbosch University.

### Intervention Setting

The public health service delivery system in 2000 was district-based with 22 out of 24 districts having one government hospital with satellite health centres. The Government District Health Officer (DHO) was charged with the oversight of services in all government facilities, and co-ordination and supervision of all health providers and programmes in the district. There were three central hospitals (at Blantyre, Lilongwe and Zomba) which were tertiary referral hospitals, but which also acted as district hospitals for their ‘host’ District.

The DHO reported directly to the central level since the introduction of health sector reform measures removed regional level management in all but the tuberculosis and EPI Programmes. The district health team had a managerial and co-ordinating role, and provided technical support and supervision.

The Ministry of Health (MOH) was entirely financed by the government and external donors. The latter provided most of the development expenditure and financed a large percentage of the recurrent costs of preventive and promotive services [9]. Free medical care in government-run facilities was the policy throughout the country.

Many districts also had a private system, the Christian Health Association of Malawi (CHAM) Hospital within their boundaries. CHAM facilities were independent, owned and run by their respective churches. However, the MOH provided CHAM facilities with staff salaries and subsidised hospitalisation costs (0.75 Malawian Kwacha per day). These facilities were also allowed to operate cost recovery from patients who could afford it.

In 2000 Malawi was the 9^th^ poorest country with a Gross National Product per capita of US$ 170 leading to chronic under-funding of health care services - total public expenditure was 9.0% [9] and only increased to 9.3% by 2005 [10]. This resulted in an on-going chronic shortage of basic health services mainly due to shortage of trained personnel, lack of skills among health professionals to manage severe and very severe pneumonia and lack of essential drugs (especially antibiotics) and supplies (such as oxygen) throughout the country [11].

The main clinical staff was paramedical Clinical Officers and Medical Assistants, supported by State Registered Nurses, State Registered Enrolled Nurses and Midwives. There was an acute shortage of all health personnel with a 39.0% vacancy rate [11]. The MOH employee/ratio to population were as follows: Doctors 1∶113,953, Registered Nurses 1∶25,857 [11]. Each of the 22 district hospitals had on average a total of 32 health personnel to manage all services within the facility. The distribution was as follows: Medical doctor 1 or none, Clinical Officers 5, Medical Assistants 4 (only worked in the outpatient department), Registered Nurses 5 and Enrolled Nurses/Midwives 17 [11]. The attrition rate of health workers due to death from HIV/AIDS at this time was as high as 41% and also a major cause of absenteeism [12].

In Malawi the ratios of doctors and nurses to population remained lower than those of its neighbouring Sub-Saharan African countries. A comparison of information available from 2004 showed lower ratios for Malawi compared with Tanzania and Zambia/ [13]: for doctors, 1.1 compared with 3.0 and 6.9; for nurses, 25.5 compared with 36.6 and 113. The WHO Standard Doctor/Population Ratio is 10∶100,000.

The intervention was carried out in the public health system in Malawi as described above. The CLHP was introduced into 24 of 25 district hospitals in Malawi. Two of these also functioned as a regional referral hospital located in a predominantly urban population while another urban-based regional referral hospital (Malawi's largest hospital based in Blantyre and also the College of Medicine's major teaching hospital) did not participate as it was better staffed and resourced. These were all non-fee paying services. Children with severe or very severe pneumonia may present at any health facility in a district but for inpatient care are referred to the dedicated paediatric wards at the district hospital according to national standards of care.

### Situational analysis

A situational analysis prior to the intervention in 2000, found that acute respiratory infection (ARI) was the second leading cause of morbidity in Malawian children below 5 years [14]. Pneumonia or lower respiratory tract infection was the most common diagnosis in hospitalized Malawian children accounting for one-quarter of paediatric admissions. Case-fatality rate for pneumonia prior to the intervention varied between 10% and 26% [15]. Problems identified included inadequate training, insufficient supplies of antibiotics, lack of adequate oxygen therapy, and lack of information to assist in planning services and procurement of essential items [15].

### Background child health indicators

In the years just prior to the intervention, Malawi reported a high under-5 mortality of 189 per 1,000 live births for the period 1996–2000.^8^ Known risk factors for frequency and severity of child pneumonia such as low birth weight (20% of live births), malnutrition (48% of children with moderate or severe stunting) and HIV infection (91,000 children living with HIV in 2006) were all highly prevalent in Malawi at the time of the intervention [16]. Immunization coverage over the intervention period was reported as relatively high - >90% for BCG and DPT3, and >80% for measles (>80%) - and there were no outbreaks of measles reported. Haemophilus influenzae type b (Hib) conjugate vaccine was introduced in 2002 with high coverage (93%) reported for 2006 [16]. Pneumococcal conjugate vaccine (PCV) was not introduced until 2011 i.e. after the implementation of the CLHP was completed.

National antenatal HIV prevalence in 2001 was estimated to be 17.1% in people 15–24 years of age [17] of age with a prevalence of up to 30% in urban settings [18], [19]. HIV prevalence among pregnant women at sentinel sites was 19.5%, 19.8% and 16.9% in 2001, 2003 and 2005. The rate of mother to child transmission in 2001 was 26.9% [20]. The estimated percentage of HIV+ pregnant women who received a full package of care to prevent mother-to-child transmission (MTCT) of HIV in 2004 was only 2.3%.

The majority of HIV-infected infants died before five years of age with pneumonia the most frequent cause of death [21]. The use of cotrimoxazole preventive therapy (CPT) for HIV-exposed infants and HIV-infected children, and the use of anti-retroviral therapy (ART) for HIV-infected children were not routinely available at the time of the intervention. Malawi received funding in 2004 from the Global Fund to Fight AIDS, Tuberculosis and Malaria to start to scale-up ART services but for children this was confined to the 3 government referral hospitals and 2 district hospitals supported by Doctors Without Borders [22].

### Intervention Design

**Figure 1:**
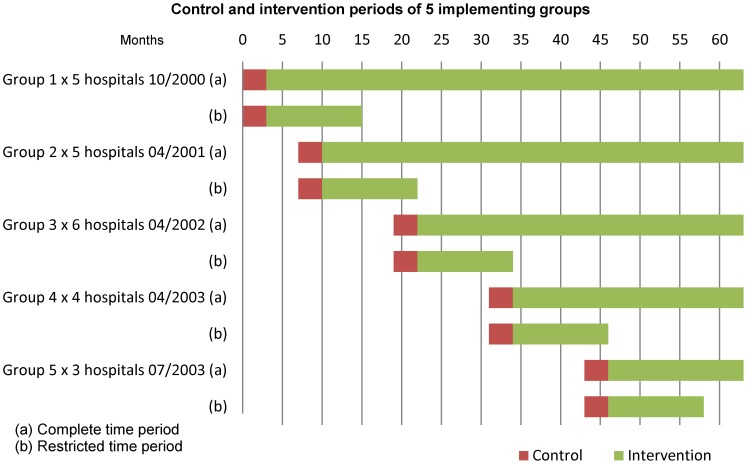
Control and intervention periods for 5 implementing groups for complete and restricted time periods.

**Figure 2:**
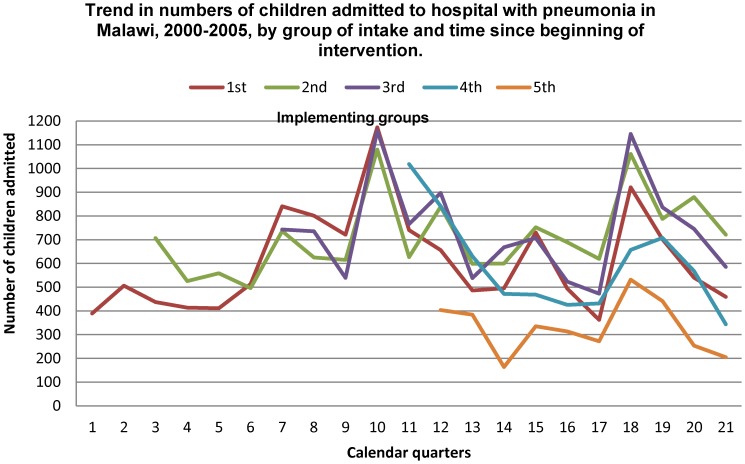
Trend in numbers of children admitted to hospital with pneumonia in Malawi, 2000–2005, by group of intake and time since beginning of intervention.

The intervention was implemented in a stepped wedge design (non-randomized). Planning commenced on the 1^st^ March 2000 and the first five district hospitals were included on the 1^st^ October 2000. Five to eight additional district hospitals were included annually in a step-wise fashion until all hospitals had been included by July 2003. Data collection ended on the 31^st^ December 2005. The sequence of hospital recruitment was chosen by the Ministry of Health according to service needs, logistical and financial reasons (Figure 1 and 2). The data recorded during the intervention were subsequently collected and analyzed for the present study from July 2011 to December 2012.

A pragmatic evaluation design was planned and was used to analyze the data, beginning with the introduction of a standardized information system that was introduced to ensure that the data generated by the health system were of good quality. Data on pneumonia admissions and outcomes were collected prospectively as part of routine care.

It was planned to use data that was collected during a pre-intervention period as the control group. The patient information forms were introduced into districts on an average of 4 months prior to implementation of the programme. Twelve districts were included and a total of 624 forms (an average 52 forms per district) were collected for analysis. A total of 373 children were admitted with a diagnosis of severe or very severe pneumonia but, of these, 123 records did not include either a diagnosis or known outcome. The remaining 250 forms contained insufficient data to ascertain if a correct diagnosis had been made.

No other reliable pre-intervention data were available. Therefore, for study purposes, it was only possible to use data collected in the first three months following implementation of the CLHP as the ‘control’ period. These data were of similar quality as that collected during the rest of the study.

### Intervention

The CLHP was based on the Union model [23] for delivering health services adapted to standard case management of the child with cough and difficult breathing [24]. The introduction of the CLHP included a substantial programme of work which included establishing an information system, quality assurance mechanisms, training, regular supervision and assurance of provision of antibiotics and oxygen to address all the various problems identified in Table 1.

**Table 1:** Summary of the situational analysis carried out prior to the implementation of the child lung health programme (CLHP), intended interventions, output indicators and outputs achieved following the CLHP intervention.

*Situation Analysis findings*	*intervention activity*	*Output indicator*	*achieved through intervention*
No hospital was implementing standard case management guidelines	All children presenting with signs of pneumonia will be managed following the CLHP technical guidelines	Children diagnosed and treated correctly as per standard categories and treatment regimens	Technical guidelines adhered to in >90% of children presenting with respiratory symptoms
Less than 10% of health workers trained in SCM of pneumonia	Health care workers from all district hospitals trained in standard case management of pneumonia	Number of health workers trained in SCM	More than 300 health care workers trained (The target exceeded by 25%
Frequent interruption of supply of antibiotics required for SCM regimens	A material management system introduced at all levels calculated on number of cases. Antibiotic reserve stock supplied	Regular uninterrupted supply of antibiotics at central, regional and district levels	No stock outs of antibiotics experienced at all levels.
No regular supply of oxygen available on paediatric wards	One designated oxygen concentrator be provided for each paediatric ward implementing CLHP	All infants <2 months admitted with severe/very severe pneumonia, all children 2–59 months admitted with very severe pneumonia or a RR≥70 received oxygen therapy	All hospitals provided with an oxygen concentrator and personnel trained at central and district level in use and maintenance.
No regular reporting system in place	Each district hospital CLHP Coordinator to fill Monthly Reports on cases and treatment outcomes and submit to central level management unit	Monthly reports on Cases of Pneumonia and on Treatment Results	99.4% achievement as of December 2005 i.e. of 2274 reports expected 2260 were received
No regular standardized supervisory/support visits being carried out by Central Level Unit	Implementation of regular standardized supervisory/support visits to implementing hospitals by Central Level Unit	Regular standardized supervisory/support visits to implementing hospitals carried out by Central Level Unit	Standardized supervision tool developed and implemented. Monthly visits carried out for 6 months then quarterly
No evaluation of ARI control activities regularly carried out	A report on external evaluation/technical support visits to the CLHP sent to the MOH every 6 months	Evaluation reports	Eight external evaluation/technical support visits plus 1 independent external review visit to the CLHP carried out and a report on each visit sent to MOH.

The essential elements of the CLHP were:

- Political commitment by the Government of Malawi to implement SCM strategies countrywide into the existing secondary health care system. This commitment implied:
  1. a structure for delivering the services
  2. no discrimination against patients in the delivery of services to promote access.
  3. sufficient financial resources for control of lung disease and other childhood illnesses
- Diagnoses and treatment based upon SCM (Table 2) with a system of quality control.
- Training of all paediatric clinical staff in SCM
- Logistics to purchase standardized drugs and to distribute them to ensure uninterrupted supplies at the management level of the District Health Office
- Recording and reporting clinical outcomes of severe and very severe pneumonia
- Supervision and evaluation of the services

**Table 2:** WHO standard case management of pneumonia defined by age groups and severity of disease.

	Standard Case Management	
Diagnosis	Presenting signs	Recommended treatment
	and symptoms	regimens
**Child 2–59 months**		
	Respiratory rate:	
**Severe pneumonia**	≥50 aged 2–11 months	**Penicillin** 50 000 units/kg IM/IV Q6h for 3 days if
	≥40 aged 12–59 months	improved then oral **amoxicillin** 25 mg/kg three times
	Lower chest wall in-drawing	daily for total of 5 to 8 days
	Respiratory rate:	
	≥50 aged 2–11 months	
	≥40 aged 12–59 months	**Chloramphenicol** 25 mg/kg IM/IV 8 hourly for 5
**Very severe**	Lower chest wall in-drawing	days if improved then three times daily for total
**pneumonia**	Cyanosis	of 10 days antibiotic treatment
	Unable to drink	
	Reduced level of consciousness	Oxygen therapy
	Severe respiratory distress	
**Infant <2 months**		
	Respiratory rate: ≥60	**Gentamicin** 7.5 mg/kg once daily for 8 days
	Severe lower chest wall in-	**Penicillin** 50 000 units/kg Q6h IM/IV for three days if
**Severe/Very severe**	drawing	improved then oral **amoxicillin** 25 mg/kg three time
**pneumonia**	Unable to breast-feed	daily for a total of 8 days antibiotic treatment
	Grunting	
	Apneic spells	Oxygen therapy
**Co-morbid conditions**		
**Pneumonia in severely malnourished child**	Signs and symptoms for severe/very severe pneumonia as above PLUS signs and symptoms for any of the following	**Cotrimoxazole** prophylaxis on admission if not acutely ill
	Marasmus	Treatment for severe or very severe pneumonia as
	Kwashiorkor	above PLUS **Gentamicin** (7.5 mg/kg IM/IV) once daily for 7 days
	<60 Weight for Height	If the child fails to improve within 48 hours, add **Chloramphenicol** (25 mg/kg IM/IV 8-hourly) for 5 days
	2–6-month-old child with central	
	cyanosis	
	Hyper-expanded chest	**Continue first-line antibiotic** (such as
	Fast breathing	**Chloramphenicol**) as mixed infection with
**Known/suspected**	Chest X-ray changes, but chest	bacteria occurs
**PcP**	clear on auscultation	
	Enlarged liver, spleen, lymph	**Oral Cotrimoxazole**: 120 mg three times daily if less
	nodes	than 5 kg; 240 mg three times daily if 5 kg or more for
	HIV test positive in mother or	21 days
	child	

Health care workers from the 24 district hospitals that provide inpatient/outpatient care for children were selected for training by each hospital administration as each of the hospitals were recruited for implementation. All paediatric clinical staff was trained as on average this meant only 3–5 nurses and one to two clinical staff per hospital. Therefor it was decided to increase this number to at least 10 and over the intervention period, a total of 312 health care workers (representing approximately 30% of all health care workers in district hospitals) to ensure more staff had received training. This included Clinical Officers (41%), State Registered Nurses (20%), Enrolled Nurses (30%), Medical Assistants (8%), other (1%). As described above there was a chronic shortage of staff and a high attrition rate in those previously trained at the recruited hospitals which meant that the new staff for these hospitals was included in the next training course. To also address this issue ongoing in-service training was provided by senior nurses and clinicians on the paediatric ward using training materials produced by the CLHP.

The training in diagnosis and treatment of children with pneumonia according to WHO SCM guidelines [24], [25] comprised an initial five day course during which theoretical and practical training occurred with a one day follow-up training one month later. Training objectives related to skills in standard case management, knowledge of child lung disease and management and planning. These included:

- General assessment of the child or young infant
- Directed history/examination, assessment and classification of a child with cough or difficult breathing
  1. assess clinical signs (e.g., respiratory rate, chest indrawing, wheeze)
  2. identify any danger signs indicating urgent care is needed
  3. assess clinical signs to determine whether pneumonia is present and if so, its severity
  4. identify other conditions and comorbidities (fever, anaemia, malnutrition, tuberculosis, malaria, asthma, HIV-related lung disease) that can be treated
  5. Identify differential diagnosis
- Prescribe appropriate treatment
- Supportive care
- Monitor child's progress
- Counselling and discharge planning
- Complete Recording Form

In addition the District Hospital Programme Coordinator was taught how to complete monthly reports and maintain adequate supply of drugs and supplies.

The training was followed by regular supervision visits to the hospitals six weeks after the training, with monthly visits for the first six months then regular three-monthly visits. On initiation of the CLHP the amount of antibiotics required for each district hospital was calculated, including one-month consumption needs plus one-month buffer stock. One year buffer stock was held at the central medical store. During supervision visits stock was checked to prevent stock-outs of antibiotics during the intervention.

Oxygen was available in a minority of district hospitals prior to the initiation of the CLHP. The CLHP acquired and installed oxygen concentrators in each district hospital paediatric ward [26].

### Patients and standard case-management

All children aged up to 59 months hospitalized with a clinical diagnosis of severe or very severe pneumonia were included. They were classified at the time of admission as severe or very severe pneumonia according to WHO recommendations. Neonates, less than two months of age, with severe and very severe pneumonia were combined in a single category. The CLHP was implemented within existing services, using treatment for pneumonia as recommended by the MoH protocols consistent with WHO guidelines [25]. Table 2 outlines the standard case management of children with pneumonia as recommended by the World Health Organization and used within the CLHP for training purposes and to guide patient care. These treatment protocols have since been updated in 2005 and 2013 [27].

### Data Collection

Demographic and clinical information was transcribed to the Hospital Inpatient Pneumonia Register, aggregated monthly and sent to a data management centre where they were entered into an EXCEL spread-sheet. The aggregated data included the following variables used in the analysis: Number of cases by age groups - <2 months, 2–11 months and 12–59 months; number of males and females; number of cases by severity – non-severe, severe and very severe and number of cases by outcome (died). There was no individual patient clinical data recorded on the monthly reports. Errors were followed up and corrected. At each supervision visit, a random sample of recording forms was checked for accuracy and consistency. (See Text S1 for supplementary material for recording and reporting).

### Statistical Analysis

Statistical analysis was that used for a cluster-randomized trial with a stepped wedge design. Five groups of hospitals were included with different starting points. The hospitals within each group started simultaneously so providing contemporaneous data. Monthly reports formed the units of analysis. The first three months after implementation in each cohort, was taken as the ‘control’ period for purposes of comparison, and each of the months following that, the intervention period. The analysis used two approaches: 1) analysis using the entire follow-up of 63 months (unrestricted) and 2) analysis of first 15 months follow-up (restricted) at each district hospital. The latter was used to limit the influence of the underlying trend in improvement of outcome occurring over time.

The primary outcome was the proportion of children who died while in hospital (case fatality rate). In addition to an implementation indicator link to intervention month, other variables included: 1) Year of the implementation; 2) Age group: <2 months, 2–11 months and 12–59 months; 3) Severity category at diagnosis: In children 2–59 months there were two categories severe and very severe which were recorded separately; 4) Region of the country: North, Central and South and 5) Season defined by month of year.

The time factor used in the analysis was month. Data within each facility were reported by this time period broken down by the covariates used in the analysis. It was then possible to recreate the individual records from the monthly report since the information reported the number of children who survived and died by unique covariate pattern. Hospitals were the clusters. All monthly records compiled at the districts were included in the analysis depending on the analysis restriction applied as outlined above.

Due to the pragmatic nature of the intervention and the stepwise implementation of the intervention it was decided to test the intervention effect in a fully adjusted model. Thus all covariates that were available from the monthly reports were included in the analysis. Interactions with the intervention were also considered. Calendar trend was extracted from the timing of the report as well as the region of the hospital.

A logistic regression model was used to estimate odds ratios and their standard errors for all the interactions of the covariates with the intervention. Of the interactions investigated only those with significant interaction between severity and intervention were retained. The clustering of children within districts was taken into account using a robust cluster variance approach. Taking only the basic design into account an overall crude intervention effect was estimated in a separate model with CLHP implementation as the only dependent variable.

As implementation began at varying times, the length of follow-up for different groups of hospitals varied, with those beginning the implementation having the longest follow-up. Therefore, the analysis was repeated (restricted analysis) using only the first 15 months follow-up at each district hospital in order to standardize the length of the intervention in the various hospitals (Figure 1). The restricted analysis became part of the model strategy to ensure contemporaneous time periods were not dominated by the intervention periods at the end of the intervention for those hospitals which started the implementation earliest. This approach was considered more objective as it limited the influence of the underlying trend in improvement of outcome that might have occurred over time in those hospitals with the longest implementation period.

## Results

Table 1 summarizes the results of the situation analysis undertaken prior to commencing implementation within the district hospitals and gives an outline of the elements of the intervention introduced by the CLHP, the output indicators used to monitor progress and the operational achievements during the course of the program. Adherence to guidelines improved from zero to over 90% among children presenting for care. Training of staff increased from less than ten per cent to over the target set for training all staff engaged in the care of small children. While interruption of essential supplies was frequent prior to the introduction of the program, it never occurred again after the program was introduced. All hospitals received the facility of oxygen supply during the implementation of the program. The poor case records and lack of routine reporting at the outset (preventing the use of any data on patient care prior to implementing the program) had been corrected with almost all (99.4%) reports being submitted by the end of the intervention period.

A total of 48 285 children were recorded in the intervention of which 1057 (2•2%) cases were admitted for non-severe pneumonia and were excluded from further analysis resulting in 47 228 cases of severe and very severe pneumonia being analysed. Of the 2274 monthly report forms generated during this period, 2260 (99•4%) were received for analysis. Figure 1 indicates the routine data that were used for analysis within the intervention. It is clear that the follow-up periods varied according to the point of introduction of the program. The follow-up period was standardized within the restricted analysis by truncating the analysis at the same point of follow-up for each of the groups of hospitals.

**Figure 3:**
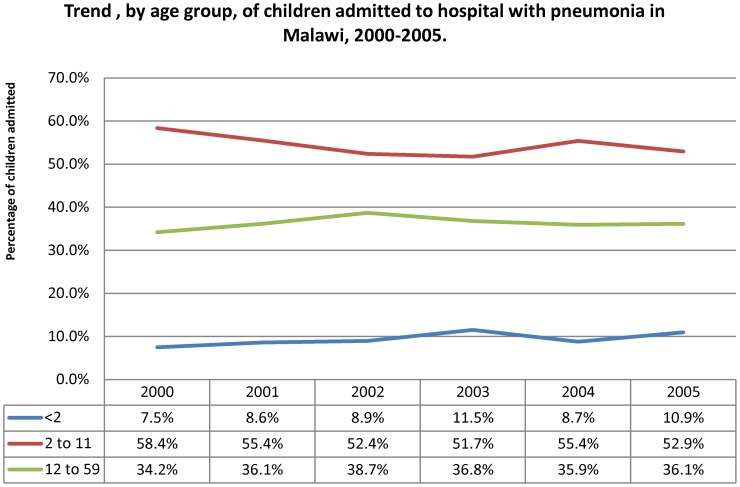
Trend by age group of children admitted to hospital with pneumonia in Malawi, 2000–2005.

**Figure 4:**
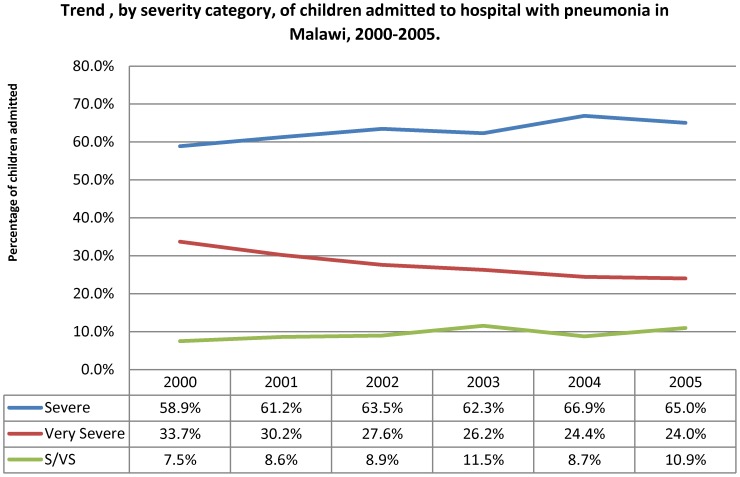
Trend by severity category of children admitted to hospital with pneumonia in Malawi, 2000–2005.

Hospitals reported a mean number of children treated for severe or very severe pneumonia of 1968 (range: 807 to 4458). Figure 2 indicates the trend in numbers of children with severe and very severe pneumonia admitted to hospital over the course of the intervention, by the hospital group of intake. The hospital intake per calendar quarter showed no significant change over the period of the intervention. The trend was similar for the proportion by age group (Figure 3) with no important change over the intervention period. There was some change in the distribution by severity grade among the children admitted to hospital (Figure 4), with a decline in the proportion of children aged two to 59 months with very severe pneumonia and a concomitant rise in the proportions classified as severe in this age group. The trend in proportions of young children (aged less than two months) showed no change over the period.

Table 3 shows the distribution of numbers of children admitted to hospital by calendar year, by age group and by classification of severity, for the entire period of follow-up and for the restricted period of follow-up. It also indicates the number and proportion of the children who died during the course of treatment. Overall, 4,605 children (9.8%) died compared with 1,600 (11.3%) when considering the more restricted period. Case fatality rate was highest for children with very severe pneumonia and was higher for younger as compared with older children. There was a steady decline in case fatality rate by calendar year overall and, to a lesser extent within the restricted period of follow-up. Deaths were equally likely to occur within the first 24 hours of hospitalization as compared with later in the course of treatment in the whole group (Table 4). The exception was the youngest age group in which a higher proportion of deaths occurred in the earlier time period. This was even more apparent for cases within the restricted period of follow-up.

**Table 3:** Numbers of children (0–59 months) treated for pneumonia by age, severity and case-fatality rate by year in district hospitals in Malawi, 2000–2005 for the restricted and unrestricted time periods.

	RESTRICTED n = 14 162			UNRESTRICTED n = 47 228		
	All	Died	CFR	All	Died	CFR
Year	Cases	No	%	cases		%
**2000**	389	73	18⋅8	389	73	18⋅8
**2001**	3558	473	13⋅3	3558	473	13⋅3
**2002**	3249	368	11⋅3	7364	771	10⋅5
**2003**	5216	560	10⋅7	12849	1225	9⋅5
**2004**	1750	126	7⋅2	9986	938	9⋅4
**2005**	-	-	-	13082	1125	8⋅6
**Total**	14162	1600	11⋅3	47228	4605	9⋅8

	RESTRICTED n = 14 162			UNRESTRICTED n = 47 228		
	All	Died	CFR	All	Died	CFR
	Cases	No.	%	Cases	No.	%
**Age group (months)** *						
<2	1386	156	11⋅3	4773	492	10⋅3
2–11	7640	1023	13⋅4	25153	2998	11⋅9
12–59	5136	421	8⋅2	17302	1115	6⋅4
**Severity grade (aged 2–59 months)** **						
severe	8727	509	5⋅8	30267	1445	4⋅8
very severe	4049	935	23⋅1	12188	2668	21⋅9

^*^ % of total number in age categories.

^**^ infants <2 months were not categorized or analyzed by separate degree of severity.

**Table 4:** Deaths before and after 24

UNRESTRICTED N = 47 228					
Characteristic	Deaths	Died<24	%	Died>24	%
**Age (months)**					
<2	492	298	60.6	194	39.4
2–11	2998	1378	46.0	1620	54.0
12–59	1115	559	50.1	556	49.9
Total	4605	2235	48.5	2370	51.5
**Severity grade**					
(2–59 months)*					
severe	1445	586	40.5	859	59.5
very severe	2668	1351	50.6	1317	49.4

RESTRICTED N = 14 162					
Characteristic	Deaths	Died<24	%	Died>24	%
**Age (months)**					
<2	156	103	66.0	53	34.0
2–11	1023	489	47.8	534	52.2
12–59	421	227	53.9	194	46.1
Total	1600	819	51.2	781	48.8
**Severity grade**					
(2–59 months)*					
severe	509	209	41.1	300	58.9
very severe	935	507	54.2	428	45.8

^*^Infants <2 months were not categorized or analyzed by separate degree of severity.

Table 5 shows the results of statistical analysis of likelihood of death, overall and for the restricted period of follow-up. Deaths were not associated with the region of the country with results not significantly different for the north, central and southern regions. There was a significant variation by calendar month with fewer deaths occurring in the middle quarters of the year. Deaths were much more likely to occur in younger children.

**Table 5:** Odds ratios from logistic regression models for mortality on the covariates including intervention period for the restricted and unrestricted analyses.

		Restricted period				Unrestricted period			
		Odds	95 CI		P	Odds	95 CI		P
Factor	Level	ratio	Lower	Upper	value*	ratio	Lower	Upper	value*
**Year**	2000	1			0⋅36	1			<0⋅001
	2001	0⋅925	0⋅64	1⋅33		0⋅98	0⋅74	1⋅28	
	2002	0⋅798	0⋅48	1⋅32		0⋅79	0⋅54	1⋅15	
	2003	0⋅704	0⋅42	1⋅18		0⋅72	0⋅51	1⋅00	
	2004	0⋅533	0⋅24	1⋅18		0⋅75	0⋅52	1⋅06	
	2005	N/A				0⋅69	0⋅5	0⋅95	
**Month**	January	1			<0⋅001	1			<0⋅001
	February	0⋅78	0⋅60	1⋅02		0⋅72	0⋅60	0⋅88	
	March	0⋅53	0⋅35	0⋅80		0⋅61	0⋅49	0⋅76	
	April	0⋅75	0⋅55	1⋅02		0⋅77	0⋅63	0⋅93	
	May	0⋅69	0⋅51	0⋅94		0⋅72	0⋅57	0⋅92	
	June	0⋅65	0⋅50	0⋅85		0⋅81	0⋅68	0⋅96	
	July	0⋅72	0⋅56	0⋅92		0⋅77	0⋅63	0⋅95	
	August	0⋅77	0⋅52	1⋅14		0⋅74	0⋅60	0⋅91	
	September	0⋅69	0⋅51	0⋅93		0⋅81	0⋅68	0⋅97	
	October	0⋅85	0⋅66	1⋅10		0⋅95	0⋅76	1⋅17	
	November	0⋅92	0⋅68	1⋅26		1⋅00	0⋅81	1⋅22	
	December	0⋅86	0⋅64	1⋅15		0⋅99	0⋅83	1⋅20	
**Region**	South	1			0⋅63	1			0⋅87
	Central	0⋅86	0⋅57	1⋅31		0⋅98	0⋅74	1⋅30	
	North	1⋅00	0⋅72	1⋅37		0⋅94	0⋅73	1⋅23	
**Age**	2–59 months	1			<0⋅001	1			<0⋅001
	2–11 months	1⋅72	1⋅41	2⋅09		1⋅86	1⋅69	2⋅05	
**Intervention by**									
**age and severity**					0⋅0305				0⋅0052
**2–59 months**									
**Severe**	pre	1				1			
	post	0⋅70	0⋅50	0⋅98	0⋅04	0⋅63	0⋅47	0⋅84	0⋅002
									
**Very severe**	pre	1				1			
	post	0⋅97	0⋅72	1⋅30	0⋅80	0⋅94	0⋅73	1⋅21	0⋅57
**<2 months** **	pre	1				1			
	post	0⋅83	0⋅56	1⋅22	0⋅34	0⋅80	0⋅57	1⋅14	0⋅20

^*^ p-value for factor.

^**^ infants <2 months were not categorized or analyzed by separate degree of severity.

Multivariate analysis of the trend in deaths over the intervention period, adjusted for age, severity, calendar month and region, showed a significant decline over the calendar years of the intervention in the overall intervention group. In comparison, although there was a decline in the likelihood of death over the intervention period, the decline was not statistically significant in the group with the restricted period of follow-up. The significant reduction in likelihood of death overall was restricted to the group of older children with severe pneumonia; there was no difference at all among the older children with very severe pneumonia or for the group of younger children. These results remained significant in the analysis of the restricted period of follow-up.

In an effort to estimate an overall crude intervention effect, a logistic regression analyses without covariates taking only the basic design into account was estimated in a separate model with CLHP implementation as the only dependent variable. This showed that in the restricted analysis the overall effect of the CLHP did not significantly decrease CFR for children admitted to hospital for pneumonia (OR = 0•82; 95%CI 0•63–1•06; p = 0•14) However in contrast to the unrestricted analysis showed there was a significant overall effect of the intervention in decreasing the CFR for all degrees of severity and age groups (OR = 0•79; 95% CI 0•64–0•99; p-value  = 0•040).

## Discussion

The trend in case fatality rates in infants and young children (1 week to 59 months of age) hospitalized and treated for severe and very severe pneumonia was evaluated over the course of the implementation of a nationwide programme to deliver standardized case management for childhood pneumonia. We were able to demonstrate the significant decline in CFR overall was no longer significant when the period of follow-up was standardized in the restricted analysis, there remained a significant decrease in CFR in children, aged 2–59 months, treated for severe pneumonia. Similarly the CFR for neonates admitted for severe/very severe pneumonia remained unchanged. An important strength of this intervention was the nation-wide implementation of the program, the prevention of antibiotics stock outs and the comprehensive and complete set of data collected over the duration of the intervention.

The high overall CFR of around 10% is within the range of that reported from nine district hospitals in Kenya but higher than the overall CFR of 6% in the study [28]. The higher case-fatality rate associated with young age and very severe pneumonia is expected and consistent with other studies [3], [29]–[31]. The lack of intervention effect in infants and children younger than 59 months suffering from very severe pneumonia is disappointing but the CFR is similar to other studies from the African region even when the studies have been conducted in central hospitals that have better resources than district hospitals [29], [31].

The introduction of the CLHP included a substantial programme of work which included establishing an information system, quality assurance mechanisms, training, regular supervision and provision of antibiotics and oxygen to address all the various problems identified in Table 1 simultaneously. Interventions that may have contributed to improved outcomes were the continuous provision of antibiotics and oxygen. Due to the planning and supervision of CLHP no shortages of antibiotics at any stage were experienced. Oxygen concentrators were acquired and installed in all district hospitals during the course of the CLHP implementation, as previously described [26]. Few district hospitals had oxygen available at the beginning of the implementation. Oxygen therapy was delivered via nasal prongs and was indicated on the basis of clinical indicators as oximetry was not available. Clinical indicators are known to be inaccurate in detecting all cases of hypoxaemia and so the use of oximetry along with supplemental oxygen could potentially have further improved outcomes [32]. The outcome of the intervention might have been influenced by the attrition of health care workers trained in the SCM of pneumonia. To minimize this effect an additional 30% more health care workers were trained.

It was not possible to determine the factors contributing to a poor outcome. There are a number of reasons that might explain a lack of benefit as seen in this analysis, especially for neonatal and very severe pneumonia in infants. First, neonates with pneumonia and infants with very severe pneumonia who are critically ill on presentation to the health services are high-risk groups for a poor outcome and often die within the first 24 hours after admission. The main impact of improved case management is likely to reduce deaths after 24 hours by clinical improvement in those that are not so critically ill at presentation, such as those with severe pneumonia.

In the HIV endemic setting, antibiotics and oxygen may not be effective against all causes of pneumonia. *Pneumocystis* pneumonia (PcP) which was common at the time of this intervention and usually fatal in Malawian infants presenting with very severe pneumonia would not have responded to first line antibiotics [30], [31]. The impact of HIV is highlighted by studies carried out in urban hospitals in South Africa showing that HIV is associated with treatment failure and poor outcomes [29]. While health workers were trained to recognise and treat PcP and other HIV-related lung disease this almost certainly did not occur as almost all participants' HIV status was recorded as unknown. Although improved HIV/AIDS services in the country would be expected to also improve outcome of treatment of children with pneumonia, the improvement in such services has not been demonstrated and therefore could not have been sufficient to explain the effect we demonstrated. Similarly, the inclusion of the 13-valent PCV into the Malawi EPI schedule since November 2011 is likely to further reduce the incidence and CFR of pneumonia in young children irrespective of HIV status [33], [34].

The intervention did show a beneficial effect in infants and children with severe pneumonia but this may have been lessened by co-morbidities or clinical overlap with pathogens that would not be responsive to standard first-line therapy, such as *Mycobacterium tuberculosis*, malaria or non-typhoidal *Salmonella*. Malawi is endemic for tuberculosis (TB), and studies from the Africa region have shown that TB is common and sometimes fatal in infants and young children with acute severe pneumonia [29]–[31], [35]. TB is likely to be under-recognised as a potential cause of acute severe pneumonia as health workers are trained to consider TB as a chronic disease associated with persistent rather than acute symptoms.

Severe malaria is common and seasonal in Malawi and can present with clinical features similar to pneumonia [36], especially severe malarial anaemia where fast breathing and chest in-drawing are common features [37]. Invasive salmonellosis is commonly associated with malarial anaemia, often presents with clinical features of pneumonia and is commonly fatal [38]. First-line antibiotics currently used for severe pneumonia in Malawi are not effective against sepsis due to non-typhoidal *Salmonella* [39]. It is notable that CFR was highest early in the rainy season and this is a peak time of year for severe malarial anaemia and invasive salmonellosis. Malnutrition is another important co-morbidity with a similar seasonal effect that could also increase the risk of poor outcome from pneumonia [40], [41]. Childhood malnutrition is very common in Malawi with high rates of stunting and wasting [42], [43].

An important limitation is that the analysis is based on routinely collected aggregate data in the district hospitals after training had been undertaken. By using the first three months of data collected after training as the baseline for comparison due to the poor quality of available data prior to the intervention is likely to have underestimated the impact of the intervention. This underestimation is supported by the high CFR (10–26%) observed during the situational analysis prior to the implementation of the CLHP [15]. The trend of improved outcomes following the first three months of intervention is noteworthy as it suggests sustained improvements in care due to this CLHP approach, rather than a temporary improvement only following training as might have been expected.

In 2005, based on the programme's success the MoH included the CLHP (within inpatient services) in the Essential Health Package funded through the Sector Wide Approach. It would have been beneficial and informative to have undertaken a formal costing but this was not possible at the time. The CLHP has been sustained beyond the cycle of external funding due to the programme's success. The CLHP has now been maintained for 8 years since the end of external project funding and is currently being expanded to 16 nongovernment hospitals. All components of the programme were still functioning well.

Improvements in child survival are being noted in many settings but consistently child pneumonia-related mortality and neonatal mortality are two of the major challenges that need to be addressed in order to reach Millennium Development Goal targets and beyond [1]. This comprehensive prospective study of the intervention to improve case-management in district hospitals in Malawi has highlighted the on-going challenges in these high mortality groups.

## Supporting Information

Text S1 **Supplementary material for recording and reporting.**
(DOCX)
