# Supplementary material for: Reducing Deaths from Severe Pneumonia in Children in Malawi by Improving Delivery of Pneumonia Case Management
Source: PLoS One. 2014 Jul 22;9(7):e102955. doi: 10.1371/journal.pone.0102955 (PMC4106861; doi:10.1371/journal.pone.0102955)
Supplement: Text S1 — Supplementary material for recording and reporting. (DOCX) [file pone.0102955.s001.docx]

**Text S1** Supplementary materials for recording and reporting

District Hospital District Registration No:

| **PNEUMONIA INPATIENT RECORDING FORM** | | | | | |  |  | | | | | |  |
| --- | --- | --- | --- | --- | --- | --- | --- | --- | --- | --- | --- | --- | --- |
| Name: | | | | | |  | Chest Xray Yes 🞏 No 🞏  If yes date taken and results: | | | | | |  |
| Address: | | | | | |  |  | | | | | |  |
| Age (months): Sex (M/F):  Number of days of signs/symptoms: More than 21 days 🞏 Less than 21 days 🞏 | | | | | |  |  | | | | | |  |
|  |  |  |  |  |  |  | Previous pneumonia in  the last 12 months Yes🞏 No 🞏  Previous hospital admissions  for pneumonia in last 12 months Yes🞏 No 🞏 | | | | | |  |
| Antibiotic treatment prior to coming to hospital: Yes 🞏 No 🞏 Self referral 🞏 Referred by Health Centre 🞏 Date of hospital admission: | | | | | |  |  |  |  |  |  |  |  |
|  | | | | | |  |  |  |  |  |  |  |  |
| Weight Kg Temperature ^0^ C Respiratory rate x 1 minute | | | | | |  |  |  |  |  |  |  |  |
| **Clinical features** | | **Classification** | **Treatment** | | | | | | | | | | |
| **Child 2 months to 5 years** |  |  | Antibiotic | Dose | Day 1 | Day 2 | | Day 3 | Day 4 | Day 5 | Day 6 | Day 7 | |
| Chest in-drawing  Severe respiratory distress  Central cyanosis  Sleepy/difficult to wake  Convulsions  Not able to breastfeed  Not able to drink  Stridor in calm child  Wheeze | Yes 🞏 No 🞏  Yes 🞏 No 🞏  Yes 🞏 No 🞏  Yes 🞏 No 🞏  Yes 🞏 No 🞏  Yes 🞏 No 🞏  Yes 🞏 No 🞏  Yes 🞏 No 🞏 Yes 🞏 No 🞏 | Very severe pneumonia 🞏  Severe pneumonia 🞏 | Benzylpenicillin |  | ⏐ ⏐ ⏐ | ⏐ ⏐ ⏐ | | ⏐ ⏐ ⏐ | ⏐ ⏐ ⏐ | ⏐ ⏐ ⏐ | ⏐ ⏐ ⏐ | ⏐ ⏐ ⏐ | |
|  |  |  | Amoxycillin |  | ⏐ ⏐ | ⏐ ⏐ | | ⏐ ⏐ | ⏐ ⏐ | ⏐ ⏐ | ⏐ ⏐ | ⏐ ⏐ | |
|  |  |  | Chloramphenicol |  | ⏐ ⏐ | ⏐ ⏐ | | ⏐ ⏐ | ⏐ ⏐ | ⏐ ⏐ | ⏐ ⏐ | ⏐ ⏐ | |
|  |  | Pneumonia 🞏  PCP 🞏  Other (specify) 🞏 | Cotrimoxazole |  | ⏐ | ⏐ | | ⏐ | ⏐ | ⏐ | ⏐ | ⏐ | |
|  |  |  | Other antibiotic  (specify) |  |  |  | |  |  |  |  |  | |
|  |  |  | Other treatment | | | | | | | | | | |
|  |  |  |  | | | | | | | | | | |
| **Young infant < 2 months** |  |  | Antibiotics | Dose | Day 1 | Day 2 | | Day 3 | Day 4 | Day 5 | Day 6 | Day 7 | |
| Chest in-drawing  Sleepy/difficult to wake  Not feeding well  Wheeze  Grunting intermittent  Grunting continuous  Stridor (calm child)  Apnoeic spells  Convulsions | Yes 🞏 No 🞏  Yes 🞏 No 🞏  Yes 🞏 No 🞏  Yes 🞏 No 🞏  Yes 🞏 No 🞏  Yes 🞏 No 🞏  Yes 🞏 No 🞏  Yes 🞏 No 🞏  Yes 🞏 No 🞏 | Very severe pneumonia/disease 🞏  Severe pneumonia 🞏  PCP 🞏  Other (specify) 🞏 | Gentamicin |  |  |  | |  |  |  |  |  | |
|  |  |  | Benzylpenicillin |  | ⏐ ⏐⏐ | ⏐ ⏐ ⏐ | | ⏐ ⏐ ⏐ | ⏐ ⏐ ⏐ | ⏐ ⏐ ⏐ | ⏐ ⏐ ⏐ | ⏐ ⏐ ⏐ | |
|  |  |  | Amoxycillin |  | ⏐ ⏐ | ⏐ ⏐ | | ⏐ ⏐ | ⏐ ⏐ | ⏐ ⏐ | ⏐ ⏐ | ⏐ ⏐ | |
|  |  |  | Other antibiotic (specify) |  |  |  | |  |  |  |  |  | |
|  |  |  | Other treatment | | | | | | | | | | |
| HIV status  Blood film (malaria) | Positive  Positive | 🞏 Negative 🞏 Unknown 🞏 Measles at this visit or in past 2 months Yes 🞏 No 🞏  🞏 Negative 🞏 Unknown 🞏 Severe malnutrition * (see below) Yes 🞏 No 🞏 | | | | | | | | | | | |

- Severe malnutrition is visible severe wasting or oedema in both feet  **please turn over**

**Hospitalisation**

| Duration of hospitalisation in either Hours Days  Admission diagnosis Discharge diagnosis |
| --- |

**Discharge and Follow-up**

| Course of antibiotics to be completed at home Yes 🞏 No 🞏  Mother informed to return with child once antibiotics completed Yes 🞏 No 🞏 | Child returned for follow-up visit Yes 🞏 No 🞏  Course of antibiotic completed****** Yes 🞏 No 🞏  Child fully recovered****** Yes 🞏 No 🞏 |
| --- | --- |

**Treatment Results**

| Treatment completed(1) 🞏 Failure at 48 hrs (2) 🞏 Failure at Day 5 🞏  Left against advise(3) 🞏 Transferred (4) 🞏 Outcome unknown (5) 🞏  Died within 24 hours of admission 🞏 Died after 24 hours of admission 🞏 **(See below for definitions)** |
| --- |

| **Additional Remarks:** |
| --- |
|  |
|  |

| **Rationale for Information/Recording System**  When the decision is reached that the child has pneumonia and requires hospitalisation then the ***“Pneumonia Inpatient Recording Form”*** must be completed in addition to other forms that may be used, such as critical care pathways. The use of this form is a prerequisite of the Project providing the drugs for treatment of such cases. The form is initiated when the patient is started on treatment and is completed on discharge. The form is provided to assist the health worker in providing good quality care for the patient. All information is transferred to the ***Pneumonia Inpatient Register.*** |
| --- |

***** If NO then tick Outcome Unknown (5) in Treatment Results section

****** If YES then child can be registered as Treatment Completed(1) in Treatment Results section

1. Course of antibiotics completed and child fully recovered
2. Treatment failure means: Worsening of fast breathing, or Worsening of chest in-drawing, or Development/persistence of abnormal sleepiness or difficulty in awakening, or development/persistence of inability to drink or poor breastfeeding.
3. Child removed from the hospital against medical advise before treatment is completed
4. Child is referred for treatment to another health facility and the result of treatment is unknown; where the result is known, that result should be recorded in place of the result "transferred
5. When mother does not return with child for follow-up visit once course of antibiotic(s) is finished

# CHILD LUNG HEALTH PROJECT

## DISTRICT HOSPITAL INPATIENT PNEUMONIA REGISTER

| Date | File No. | Name in Full | Address  Village/District | Age  in  Months | Sex | Previous  Pneumonia | Previous  Admission | Clinical Signs of Pneumonia | | | | | Diagnosis | | | |  |
| --- | --- | --- | --- | --- | --- | --- | --- | --- | --- | --- | --- | --- | --- | --- | --- | --- | --- |
|  |  |  |  |  | M/F | in last  12 months  Y/N | for  Pneumonio  Y/N | RespiratoryRate x  1 Minute | Chest  In -drawing | Not Drinking/Feeding | Central  Cyanosis | Other Major Signs (Specify) | Pneu-  monia | Severe Pneu- monia | Very Severe Pneumonia/ Disease | PCP |  |
| **----** |  |  |  |  |  |  |  |  |  |  |  |  |  |  |  |  |  |
| **------** |  |  |  |  |  |  |  |  |  |  |  |  |  |  |  |  |  |
| **------** |  |  |  |  |  |  |  |  |  |  |  |  |  |  |  |  |  |
| **------** |  |  |  |  |  |  |  |  |  |  |  |  |  |  |  |  |  |
| **------** |  |  |  |  |  |  |  |  |  |  |  |  |  |  |  |  |  |
| **------** |  |  |  |  |  |  |  |  |  |  |  |  |  |  |  |  |  |
| **------** |  |  |  |  |  |  |  |  |  |  |  |  |  |  |  |  |  |
| **------** |  |  |  |  |  |  |  |  |  |  |  |  |  |  |  |  |  |
| **------** |  |  |  |  |  |  |  |  |  |  |  |  |  |  |  |  |  |
| **------** |  |  |  |  |  |  |  |  |  |  |  |  |  |  |  |  |  |
| **------** |  |  |  |  |  |  |  |  |  |  |  |  |  |  |  |  |  |
| **------** |  |  |  |  |  |  |  |  |  |  |  |  |  |  |  |  |  |
| **------** |  |  |  |  |  |  |  |  |  |  |  |  |  |  |  |  |  |
| **------** |  |  |  |  |  |  |  |  |  |  |  |  |  |  |  |  |  |

| Associated Conditions | | Diagnosis  Confirmed  by Xray  Y/N | HIV Status | | | Admitted | | Antibiotics for Pneumonia | | | | | Addition treatment | Treatment Results | | | | | | |
| --- | --- | --- | --- | --- | --- | --- | --- | --- | --- | --- | --- | --- | --- | --- | --- | --- | --- | --- | --- | --- |
| Respir-  atory | Other(s)  (Specify |  | P  o  s | N  e  g | Un  known | Y  e  s | N  o  **(1)** | Benzyl-  penicillin | Chloram-  phenicol | Genta-  micin | Amoxy  -cillin | Other(s)  (Specify) | Specify | Treatment  Completed  **(2)** | Failure  48 hrs  **(3)** | Failure  5 days  **(3)** | Left against advise  **(4)** | Trans-  ferred  **(5)** | Died  before  24 hrs | Died  after  24 hrs |
| **------------** |  |  |  |  |  |  |  |  |  |  |  |  |  |  |  |  |  |  |  |  |
| **------------** |  |  |  |  |  |  |  |  |  |  |  |  |  |  |  |  |  |  |  |  |
| **------------** |  |  |  |  |  |  |  |  |  |  |  |  |  |  |  |  |  |  |  |  |
| **------------** |  |  |  |  |  |  |  |  |  |  |  |  |  |  |  |  |  |  |  |  |
| **------------** |  |  |  |  |  |  |  |  |  |  |  |  |  |  |  |  |  |  |  |  |
| **------------** |  |  |  |  |  |  |  |  |  |  |  |  |  |  |  |  |  |  |  |  |
| **------------** |  |  |  |  |  |  |  |  |  |  |  |  |  |  |  |  |  |  |  |  |
| **------------** |  |  |  |  |  |  |  |  |  |  |  |  |  |  |  |  |  |  |  |  |
| **------------** |  |  |  |  |  |  |  |  |  |  |  |  |  |  |  |  |  |  |  |  |
| **------------** |  |  |  |  |  |  |  |  |  |  |  |  |  |  |  |  |  |  |  |  |
| **------------** |  |  |  |  |  |  |  |  |  |  |  |  |  |  |  |  |  |  |  |  |
| **------------** |  |  |  |  |  |  |  |  |  |  |  |  |  |  |  |  |  |  |  |  |
| ------------ |  |  |  |  |  |  |  |  |  |  |  |  |  |  |  |  |  |  |  |  |
| ------------ |  |  |  |  |  |  |  |  |  |  |  |  |  |  |  |  |  |  |  |  |

1 If "No" due to admission refused indicate with "**R**"

2 Course of antibiotics completed and child fully recovered

1. Treatment failure means: Worsening of fast breathing, or Worsening of chest in-drawing, or Development/persistence of abnormal sleepiness or difficulty in awakening, or Development/persistence of inability to drink or poor breastfeeding.

4 Child removed from the hospital against medical advise before treatment is completed

5 Child is referred for treatment to another health facility and the result of treatment is unknown; where the result is known, that result should be recorded in place of the result "transferred"

CHILD LUNG HEALTH PROJECT **MONTHLY REPORT ON CASES OF PNEUMONIA**

| Name of District  Name of Hospital  Patients registered in MONTH | District Coordinator's Name  Signature  Date |
| --- | --- |

|  | INFANTS (under 2 months old) | | CHILD (2 to 59 months) | | | | | | Total |
| --- | --- | --- | --- | --- | --- | --- | --- | --- | --- |
| Gender | Severe pneumonia | Very severe | Pneumonia | | Severe pneumonia | | Very severe pneumonia | | by |
|  |  | pneumonia/disease | 2 - 11  months | 12 – 59 months | 2 - 11 months | 12 – 59 months | 2- 11 months | 12 – 59 months | gender |
| Males |  |  |  |  |  |  |  |  |  |
| Females |  |  |  |  |  |  |  |  |  |
| Total by severity of pneumonia |  |  |  |  |  |  |  |  |  |

**Definitions to use when completing the form:**

**Severity of pneumonia**

**YOUNG INFANT 1 to 8 weeks):**

Severe pneumonia a patient with cough or difficult breathing who presents with fast breathing and/or chest indrawing

Very severe a patient with cough or difficult breathing who presents with fast breathing and/or chest indrawing and with any one or more of the following:

pneumonia/disease not feeding well, convulsions, abnormally sleepy or difficult to wake, wheezing, raised temperature (>38 ^o^) or low temperature (< 35.5^o^), central cyanosis, grunting or apnoeic episodes.

**CHILD (2-59 months old):**

Pneumonia a patient presenting with cough or difficult breathing with fast breathing

Severe pneumonia a patient presenting with cough or difficult breathing with fast breathing and/or chest indrawing

Very severe pneumonia a patient with severe pneumonia with any one or more of the following: central cyanosis, severe respiratory distress, not feeding/drinking well, convulsions, abnormally sleepy or difficult to wake, stridor in a calm child.

CHILD LUNG HEALTH PROJECT

**MONTHLY REPORT OF PNEUMONIA TREATMENT RESULTS**

(REGISTERED IN THE HOSPITAL IN THE PREVIOUS MONTH)

| Name of District__________________________________________________________  Name of Hospital__________________________________________________________  Patients registered in _____ Month/year______ | Hospital Coordinator__________________________________  Signature_______________________________________________  Date_____________ |
| --- | --- |

| Type of case | Antibiotic given | Total no: of pneumonia patients registered in above month | | Completed Treatment | | Left against advise | | Trans ferred | | Outcome unknown | | Treatment Failures | | | | Died | | | | |
| --- | --- | --- | --- | --- | --- | --- | --- | --- | --- | --- | --- | --- | --- | --- | --- | --- | --- | --- | --- | --- |
|  |  |  |  |  |  |  |  |  | |  | | 48 hrs | | 5 days | | Before 24 hrs | | | After 24 hrs | |
| **Young infants**: (1 through 8 weeks) | Benzylpenicillin/Gentamicin/ Amoxicillin |  | |  | |  | |  | |  | |  | |  | |  | | |  | |
| Severe pneumonia |  |  | |  | |  | |  | |  | |  | |  | |  |  |  |  |  |
| And  Very severe Pneumonia/disease | Other(s) (specify): |  | |  | |  | |  | |  | |  | |  | |  | | |  | |
|  |  |  | |  | |  | |  | |  | |  | |  | |  |  |  |  |  |
| **Children:** (2 through 59 months) |  | 2 to 11 | 12 to 59 | 2 to 11 | 12 to 59 | 2 to 11 | 12 to 59 | 2 to 11 | 12 to 59 | 2 to 11 | 12 to 59 | 2 to 11 | 12 to 59 | 2 to 11 | 12 to 59 | | 2 to 11 | 12 to 59 | 2 to 11 | 12 to 59 |
| Pneumonia | Cotrimoxazole |  |  |  |  |  |  |  |  |  |  |  |  |  |  | |  |  |  |  |
|  | Other(s) (specify): |  |  |  |  |  |  |  |  |  |  |  |  |  |  | |  |  |  |  |
| Severe pneumonia | Benzylpenicillin/ Amoxicillin |  |  |  |  |  |  |  |  |  |  |  |  |  |  | |  |  |  |  |
|  | Other(s) (specify): |  |  |  |  |  |  |  |  |  |  |  |  |  |  | |  |  |  |  |
| Very severe pneumonia | Chloramphenicol |  |  |  |  |  |  |  |  |  |  |  |  |  |  | |  |  |  |  |
|  | Other(s) (specify): |  |  |  |  |  |  |  |  |  |  |  |  |  |  | |  |  |  |  |

**please turn over for definitions to use when completing the form**

**Definitions to use when completing the form:**

**Severity of pneumonia**

| **YOUNG INFANT (1 to 8 weeks):**  Severe pneumonia  and very severe pneumonia/disease | a patient with cough or difficult breathing who presents with fast breathing and/or chest indrawing and with any one  or more of the following:  not feeding well, convulsions, abnormally sleepy or difficult to wake, stridor in a calm child, wheezing,  raised temperature (>38 ^o^) or low temperature (< 35.5^o^), central cyanosis, grunting or apnoeic episodes. |
| --- | --- |
| **CHILD (2-59 months old):**  Pneumonia  Severe pneumonia  Very severe pneumonia | a patient presenting with cough or difficult breathing with fast breathing  a patient presenting with cough or difficult breathing with fast breathing and/or chest indrawing  a patient with severe pneumonia who has central cyanosis and/or is unable to drink |

**Treatment results**

Treatment completed Course of antibiotics completed i.e. all prescribed injections/tablets/capsules have been given and the young infant/child is fully recovered i.e. respiratory rate, temperature and drinking/eating/feeding pattern, for the particular child, have returned to normal.

Treatment failure Failure of initial antibiotic treatment/antibiotic was changed because of: Worsening of fast breathing, **or** Worsening of chest in-drawing, **or** Development/persistence of abnormal sleepiness or difficulty in awakening, **or** development/persistence of inability to drink or poor breastfeeding. It should be recorded if this was at 48 hours or 5 days.

Left against advise Child removed from the hospital against medical advice before treatment is completed

Transferred Child is referred for treatment to another health facility and the result of treatment is unknown; where the result is known, that result should be recorded in place of the result "transferred".

Outcome unknown When mother does not return with child for follow-up visit once course of antibiotic(s) is finished

Died When a child dies during treatment it should be recorded whether the child died during or after the first 24 hours

following admission
